# Observations on the effect of enteral nutrition support on serum nutritional proteins, proinflammatory cytokines and immunoglobulins in patients with cerebral hemorrhage

**DOI:** 10.5937/jomb0-58247

**Published:** 2025-11-05

**Authors:** Wenjun Tan, Xiaoqiang Li, Xiaolu Tang

**Affiliations:** 1 The First Affiliated Hospital of Xi'an Jiaotong University, Department of Surgery Intensive Care Unit, Xian, Shaanxi, 710061, China; 2 The Second Affiliated Hospital of Xi'an Medical College, Department of Neurosurgery, Xian, Shaanxi, 710038, China

**Keywords:** cerebral haemorrhage, enteral nutrition support, serum nutritional proteins, proinflammatory cytokines, immunoglobulins, cerebralno krvarenje, enteralna nutritivna podrška, serumski nutritivni proteini, proinflamatorni citokini, imunoglobulini

## Abstract

**Background:**

This study will analyse the changes in serum nutrient protein, a proinflammatory cytokine, and immunoglobulins before and after enteral nutrition support (ENS) in patients with intracerebral haemorrhage (ICH), which will serve as a reference for future clinics when performing ENS.

**Methods:**

This retrospective observational study included 160 patients with ICH (76 in the intermittent group and 84 in the continuous group), and changes in indicators before and after EN intervention were retrospectively analysed in both groups, including serum nutrient protein (albumin ALB, transferrin TRF, prealbumin PAB), proinflammatory cytokine (IL-1 b, IL-6, TNF-a), immunoglobulins (IgA, IgG, IgM) and gastrointestinal tolerance.

**Results:**

There was no difference in adverse reactions between the two groups during ENS (P&gt;0.05). After ENS, ALB, TRF PAB, IgA, IgG, and IgM were significantly increased in both groups, while IL-1 b, IL-6, and TNF-a were decreased (P&lt;0.05). After ENS, there was no difference in serum nutrient protein between the two groups (P&gt;0.05). Still, proinflammatory cytokines were lower in the intermittent group than in the continuous group, while immunoglobulins were higher than in the intermittent group (P&lt;0.05).

**Conclusions:**

ENS exerts neuroprotection through the "intestinal barrier repair-immune remodeling-inflammation inhibition axis".

## Abstract (sr)

**Uvod:**

Ova studija ima za cilj da analizira promene u nivoima serumskih nutritivnih proteina, proinflamatornih citokina i imunoglobulina pre i posle enteralne nutritivne podrške (ENS) kod pacijenata sa intracerebralnim krvarenjem (ICH), kako bi se obezbedila referentna osnova za buduću kliničku praksu u primeni ENS.

**Metode:**

U ovu retrospektivnu opservacionu studiju je bilo uključeno 160 pacijenata sa ICH (76 u intermitentnoj grupi i 84 u kontinuiranoj grupi). Retrospektivno su analizirane promene u parametrima pre i posle EN intervencije u obe grupe, uključujući serumske nutritivne proteine (albumin - ALB, transferin - TRF, prealbumin - PAB), proinflamatorne citokine (IL-1 b, IL-6, TNF-a), imunoglobuline (IgA, IgG, IgM) i gastrointestinalnu toleranciju.

**Rezultati:**

Nije bilo značajne razlike u neželjenim reakcijama između grupa tokom ENS (P&gt; 0,05). Nakon ENS, ALB, TRF, PAB, IgA, IgG i IgM su bili značajno povećani u obe grupe, dok su IL-1 b, IL-6 i TNF-a bili smanjeni (P&lt; 0,05). Nakon ENS, nije bilo razlike u nivoima serumskih nutritivnih proteina između grupa (P&gt; 0,05). Ipak, nivo proinflamatornih citokina bio je niži u intermitentnoj grupi nego u kontinuiranoj, dok su nivoi imunoglobulina bili viši u intermitentnoj grupi (P&lt; 0,05).

**Zaključak:**

Enteralna nutritivna podrška ostvaruje neuroprotectivni efekat delovanjem kroz osovinu "obnova crevne barijere - imunološko preoblikovanje - suzbijanje upale".

## Introduction

Intracerebral haemorrhage (ICH), a condition with the highest mortality rate among acute cerebrovascular diseases, refers to the bleeding caused by non-traumatic intraparenchymal vascular rupture, accounting for 20 to 30 per cent of all strokes, with a mortality rate of 30 to 40 per cent in the acute phase [1]. Most patients with ICH have concomitant hypertension, with hypertension associated with arteriolosclerosis being the most common inducement [2]. ICH usually occurs in men aged 50-70 and is prone to occur in winter and spring, with no warning before bleeding [3]. At present, clinical treatment is mainly based on conservative regimens, such as controlling bleeding, reducing intracerebral pressure, and protecting brain tissue around hematoma. However, for most patients with acute onset of ICH, conventional conservative treatment cannot achieve the ideal effect, and surgical intervention is required to complete more direct intervention treatment purposes [4]. It is well known that the body of patients after ICH is in a hypermetabolic state, where nutritional imbalance and inflammatory response are intertwined, exacerbating secondary brain damage and affecting prognosis [5]. For patients with ICH, enteral nutrition support (ENS) has gradually become one of the essential links in their clinical treatment [6]. ENS, as a key intervention, not only maintains nitrogen balance by supplementations of serum nutrient protein (such as albumin and prealbumin) but also may regulate the release of proinflammatory cytokines (such as IL-6 and TNF-α). Reduce the damage of »inflammatory storm« to nerve function [7] [8]. At the same time, immunoglobulin (IgG, IgA, etc.) is the core component of humoral immunity, and its level changes can reflect the immune status and infection risk of patients. In contrast, nutritional status directly affects its synthesis efficiency [9].

However, studies on the dynamic association of serum nutrient protein, proinflammatory cytokine and immunoglobulin after ENS intervention in ICH patients are still limited, and how the three synergistically affect the disease process is not clear. In this study, we will analyse the changes in the above indicators before and after ENS, aiming to reveal the regulatory effect of nutritional intervention on the metabolic-immune-inflammatory network in ICH patients and provide a theoretical basis for optimising clinical nutritional strategies.

## Materials and methods

### Study subjects

The sample size was calculated using GPower (version 3.1) based on a detectable effect size of 0.3 (α = 0.05, (β = 0.2), requiring 160 patients. A retrospective analysis was conducted on 160 ICH patients who received treatment at two class A tertiary hospitals from October 2021 to October 2023. Among them, 76 patients received intermittent ENS (intermittent group), and 84 patients received continuous ENS (continuous group). Intermittent ENS: 4-hourly boluses; Continuous ENS: 24-hour infusion via the pump. The study was conducted in strict accordance with the Declaration of Helsinki, and all subjects signed informed consent. Approved by the Ethics Committee of the Second Affiliated Hospital of Xi'an Medical College.

### Criteria for patient enrollment and exclusion

Inclusion criteria: Patients over 18 years old were diagnosed as ICH by our hospital and met the clinical symptoms of ICH [10], with complete clinical data, hospital admission within 24 hours after onset, no contraindications to enteral nutrition, and informed consent signed by themselves or their family members. The patient was admitted to the hospital and treated by the same surgical team using minimally invasive punctual drainage. Sedation and pain management with midazolam and opioids. Exclusion criteria: Patients with vascular malformation, hemangioma, intracranial space-occupying lesion, or cerebral hernia were excluded; those with diseases that seriously affect nutritional metabolism, haematological diseases, immune system diseases, or major organ failure (defined as 2 organ dysfunctions according to the Sequential Organ Failure Assessment [SOFA] score) were also excluded.

### Sample collection and testing

Fasting venous blood was collected before and 7 days after nutritional support, and centrifugation (3000 rpm/min) was performed for 15 min after 30 min of standing at room temperature to separate the serum, which was divided into 3 parts. Albumin (ALB), transferrin (TRF), prealbumin (PAB), haemoglobin (Hb), and retinol-binding protein (RBP) were measured using an automatic biochemical analyser (BS-1000M, Mairey, Shanghai, China), interleukin (IL)-1β, IL-6, and tumour necrosis factor-α (TNF-α) were determined following the enzyme-linked immunosorbent assay kit instructions. The kits were purchased from Beijing Boao Sen Biotechnology Co., Ltd., and the operation process was carried out in strict accordance with the instructions for the kits. The ELISA kits had a sensitivity of <10 pg/mL and intra-/inter-assay coefficients of variation of <8% and <10%, respectively. Scattering turbidimetric assay for the detection of immunoglobulin IgA, IgG, and IgM: Serum samples to be tested are mixed with latex particles of reagent encapsulated with specific antihuman IgA/IgG/IgM antibodies, and the antigen-antibody binding forms an immune complex, increasing the turbidity of the solution. A fully automated biochemical analyser was used to measure the change in light scattering intensity within the reaction system, with the amount of light scattered proportional to the amount of complex. The immunoglobulin concentration in the sample is calculated by means of a preset standard curve (drawn using standards of known concentration).

### Feeding intolerance

Feeding intolerance can be diagnosed when one or more conditions, such as vomiting, diarrhoea, abdominal distension, gastric retention, and gastrointestinal bleeding, occur.

### Statistical methods

SPSS23.0 statistical software (IBM Corp., Armonk, NY, USA) was used to analyse the data. Qualitative data were represented by [n(%)], and the comparison adopted the χ^2^ test. Normality was assessed using the Shapiro-Wilk test. (x̄±s) was used to describe the quantitative data, the inter-group and intra-group comparisons of which employed the independent sample t-test and paired t-test, respectively. *P*<0.05 was the significance threshold.

## Results

### Comparison of clinical baseline data

First of all, the age, gender, body mass index, and other data of the two groups were compared, and no statistical significance was identified between them (*P*>0.05), confirming comparability. There was no difference in the incidence of gastrointestinal feeding intolerance between the two groups (*P*>0.05) (Table 1).

**Table 1:** Comparison of clinical baseline data and gastrointestinal feeding intolerance.

Groups		Intermittent group	Continuous group	χ^2^/t	*P*
Age (years)		60.6±2.6	60.9±3.2	0.647	0.519
Sex male/female		41 (53.9)/35 (46.1)	45 (53.6)/39 (46.4)	0.002	0.962
Body mass index (kg/m^2^)		25.2±3.2	25.5±2.0	0.718	0.474
Operating time (min)		50.6±7.2	49.7±9.0	0.666	0.506
Cigarette smoking	yes/no	46(60.5)/30(39.5)	50 (59.5)/34 (40.5)	0.017	0.897
Family history of illness	yes/no	46(60.5)/30(39.5)	79 (94.1)/5 (5.9)	0.036	0.85
Vomiting (%)		3 (3.9)	2 (2.2)		
Diarrhea (%)		1(1.3)	2 (2.2)		
Bloating (%)		2 (2.6)	2 (2.2)		
Gastric retention (%)		0 (0.0)	1 (1.2)		
Gastrointestinal bleeding (%)		1(1.3)	0 (0.0)		
Incidence of total intolerance (%)		7 (9.2)	7 (8.1)	0.087	0.768

### Comparison of nutritional status

**Figure 1:**
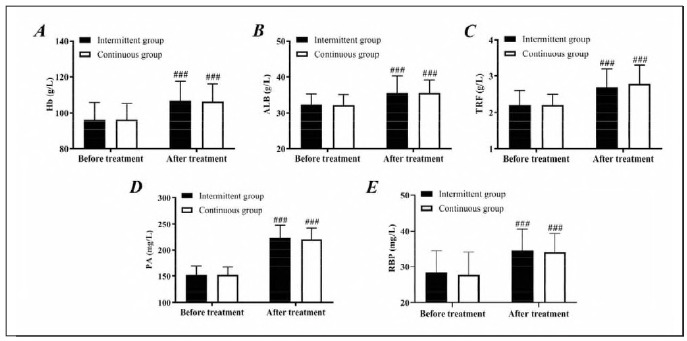
Comparison of nutritional status.<br>A: Comparison of Hb. B: Comparison of ALB. C: Comparison of TRF. D: Comparison of PA. E: Comparison of RBP compared with before treatment, ###P<0.001.

The two groups were not statistically different in nutritional status before treatment (*P*>0.05). After treatment, the levels of HGB, ALB, TRF, PA, and RBP of both groups elevated (*P*<0.05). However, the differences between the two groups were still not statistically significant (*P*>0.05), indicating the excellent effects of both kinds of ENS on improving the nutritional status of ICH patients (Figure 1).

### Comparison of proinflammatory cytokine

**Figure 2:**
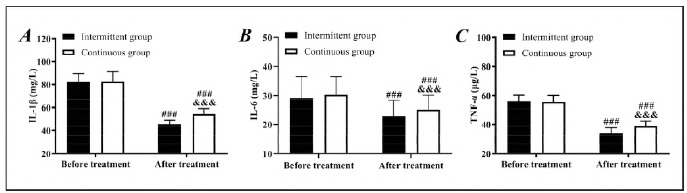
Comparison of proinflammatory cytokine.<br>A: Comparison of IL-1β. B: Comparison of IL-6. C: Comparison of TNF-α, compared with before treatment, ###P<0.001, compared with the intermittent group, &&&P<0.001.

Similarly, the two groups showed no significant difference in the detection results of proinflammatory cytokine before treatment (*P*>0.05). After treatment, the levels of IL-1β, IL-6, and TNF-α in both groups decreased, with even lower levels in the intermittent group (*P*<0.05), suggesting that intermittent ENS has a better anti-inflammation effect (Figure 2).

### Comparison of immune function

**Figure 3:**
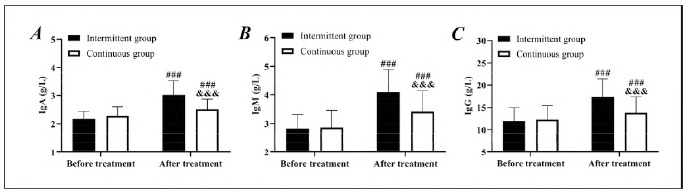
Comparison of immune function.<br>A: Comparison of IgA. B: Comparison of IgM. C: Comparison of IgG, compared with before treatment, ###P<0.001, compared with the intermittent group, &&&P<0.001.

No notable difference was determined in pretreatment IgA, IgM, and IgG levels between groups (*P*>0.05). At the same time, the intermittent group showed higher IgA, IgM, and IgG levels than the continuous group after treatment (*P*<0.05). In both groups, a marked increase in IgA, IgM, and IgG was observed after treatment (*P*<0.05). It is suggested that intermittent ENS can better improve the immune function of patients (Figure 3).

## Discussion

In this study, we found no difference in the incidence of gastrointestinal feeding intolerance between the two nutritional support programs, confirming that both ENS regimens are reliable for gastrointestinal health in ICH patients. Changes in patients' nutritional status are also one of the key links in ENS [11]. In this study, we found that the nutritional status of the continuous and intermittent groups did not change significantly after treatment. Still, the immune function and proinflammatory cytokine of the intermittent group were more obviously improved, indicating that intermittent ENS has higher clinical application value in improving the overall nutritional status of ICH patients. However, we found significant improvements in serum nutrient protein and immunoglobulins and reduced levels of proinflammatory cytokine after ENS in both groups, and these findings corroborate the importance of ENS for the recovery of ICH patients.

In response to the changes in serum nutrient protein, proinflammatory cytokine, and immunoglobulins in ICH and ENS, we believe that the mechanism of action can be attributed to the following three levels of deep interaction:

- (1) Serum nutrient protein: a bridge from metabolic support to inflammation-immunomodulation. ALB and PA white are not only indicators of nutrient reserves in the traditional sense but their functions after ICH go beyond mere nitrogen balance maintenance [12] [13]. The mechanism of elevated albumin levels after EN intervention in this study may include the following: Hypoalbuminemia can exacerbate cerebral oedema of vascular origin, whereas albumin reduces the degradation of tight junction proteins (e.g., ZO-1) between endothelial cells of the blood-brain barrier (BBB) by maintaining plasma colloidal osmolality, which inhibits the expansion of oedema [14]. In addition, thiol groups on the surface of the ALB molecule directly neutralise oxygen radicals (ROS) and inhibit oxidative stress driven by the Fenton reaction by binding to heme and iron ions released from hematomas [15]. A study by Komara NL et al. also demonstrated that the ability of ALB to bind to gut-derived endotoxin (LPS) reduces systemic inflammatory response syndrome (SIRS) [14]. The results of Manna D et al. [16] mentioned that ALB reduces the release of proinflammatory cytokines such as IL-6, TNF-α and other proinflammatory cytokines by inhibiting NF-κB nuclear translocation and down-regulating the activation of TREM-1/TLR4 pathway in microglia. We hypothesised that this process may be related to the metabolic regulation of fatty acid components (e.g., arachidonic acid) bound to ALB [17].
- (2) Proinflammatory cytokine: Targeted intervention of ENS on the »neuro-immune-gut axis«. Brain parenchymal injury after ICH triggers microglia M1 polarisation, releasing large amounts of IL-1β, IL-6, and TNF-α, forming an »inflammatory storm« [18]. In the present study, we found that ENS significantly reduced the levels of the proinflammatory cytokine. The mechanism may involve the glutamine provided by ENS, which is the main energy source of intestinal epithelial cells. It promotes the synthesis of tight junction proteins by activating the mTORC1 pathway, reduces the translocation of intestinal bacteria and the entry of LPS into the bloodstream, and thus inhibits the activation of the TLR4/MyD88 signalling pathway in Kupffer cells [19]. ω-3 polyunsaturated fatty acids (e.g., EPA, DHA) in the ENS formulation inhibited NLRP3 inflammatory vesicle assembly via a PPAR -dependent pathway while promoting macrophage conversion to an anti-inflammatory M2 phenotype [20]. In addition, branched-chain amino acids (BCAAs) inhibit IKKβ phosphorylation and block NF-κB inflammatory signalling through activation of the AMPK pathway [21]. ENS stimulates the release of cholecystokinin (CCK) from enteroendocrine cells, enhances the α7 nicotinic acetylcholine receptor (α7nAChR)-mediated cholinergic anti-inflammatory pathway via vagal afferent signalling, and directly inhibits TNF-α synthesis in splenic macrophages [22].
- (3) Immunoglobulins: humoral immune remodelling under nutrient-microbiota interactions. Secondary immunosuppression (e.g., decreased IgG and IgA levels) after ICH is closely associated with an increased risk of infection [23]. It has been mentioned that butyric acid enhances Foxp3 gene expression by inhibiting histone deacetylase (HDAC), promotes regulatory T cell (Treg) expansion, and indirectly inhibits the toxic effects of excessive inflammation on B cells [24]. This may be the mechanism of the effect of ENS on immune function.

However, as this study is a retrospective analysis with a small sample size and a short follow-up period, the results may be biased, which needs to be confirmed by a large-sample multi-center study. The 7-day follow-up period limits insights into the long-term effects of ENS on immunomodulation. We need longer follow-ups to refine the effect of ENS on immune function in patients with ICH. Subsequently, while preclinical studies support ALB's role in BBB integrity, further validation using ZO-1 ELISA or Western blot is warranted. Future studies should incorporate flow cytometry to analyse Treg/Th17 cell dynamics during ENS.

## Conclusion

The role of ENS in ICH patients is far beyond the scope of traditional nutritional supply, which constitutes a core mechanism of neuroprotection by improving the inflammatory response of patients and the multistage connection of immune function. Future studies should integrate multi-omics approaches to explore individualised nutritional strategies.

## Dodatak

### Availability of data and materials

Original data in this study are available from the corresponding author upon a reasonable request.

### Conflict of interest statement

All the authors declare that they have no conflict of interest in this work.
